# High power ultrafast phase-locked laser at 2060 nm from a doubly resonant optical parametric oscillator

**DOI:** 10.1038/s41598-026-40002-x

**Published:** 2026-02-18

**Authors:** Han Rao, Robin Mevert, Fridolin Jakob Geesmann, David Zuber, Ayhan Demircan, Ihar Babushkin, Uwe Morgner

**Affiliations:** 1 Leibniz University Hannover, Institute of Quantum Optics, Hannover, Germany; 2 Cluster of Excellence PhoenixD, Hannover, Germany; 3 Max Born Institute, Berlin, Germany

**Keywords:** Engineering, Optics and photonics, Physics

## Abstract

A high-power 2-$\mu$m phase-locked femtosecond source is demonstrated by a degenerate doubly resonant optical parametric oscillator (DROPO), which is synchronously pumped by a home-built Yb:YAG Kerr-lens mode-locked thin-disk laser. A dither-free scheme, incorporating an intracavity ’parasitic’ sum-frequency of signal and pump as an error signal, has been used to stabilize the DROPO at degeneracy. To our knowledge, with a pump power of 15.8 W, this system achieves the highest output power (5.6 W) and conversion efficiency (35 %) for an actively stabilized, degenerate BBO-based DROPO operating at 2 $\mu$m. The long-term stability measurement of the power over 90 minutes shows a root mean square (RMS) power noise of 0.78%, demonstrating excellent stability and reliable performance.

## Introduction

Optical frequency combs have attracted significant interest over the past few decades, driving the development of various applications^1–3^, particularly in the field of precision optical frequency metrology. These developments notably contributed to the awarding of the 2005 Nobel Prize in Physics to John Hall and Theodor Hänsch^4^. Beyond their metrological power, frequency-comb-based laser sources –particularly those operating around 2 $\mu$m – have become indispensable in areas ranging from ultrafast dynamics to remote sensing and medical diagnostics^5–7^. High-power, low-noise pulses in this spectral region not only enable efficient pumping of mid-infrared parametric sources^8,9^, but also drive strong nonlinear interactions^10^. Among these, the efficient generation of Brunel radiation in the THz range is of particular interest, as it relies critically on the precise phase relationship between two-color driving fields. Our self-phase-locked degenerate DROPO offers a unique solution, providing intrinsically phase-locked two-color fields at 2 $\mu$m and 1 $\mu$m, ideally suited for controlled studies of Brunel radiation.

One of the most effective approaches to generate coherent light in the 2-$\mu$m region is through nonlinear down-conversion of shorter-wavelength combs in synchronously pumped OPOs^11^. In a DROPO, stable operation requires that both signal and idler waves resonate simultaneously with the pump in the cavity. This condition is highly sensitive to even tiny cavity-length fluctuations, due to the dispersive nature of the system. Such perturbations can easily disturb the longitudinal mode matching and phase stability needed for resonance^12^. At exact degeneracy, however, the signal and idler become indistinguishable, and the DROPO naturally enforces a self-phase-locked state with the pump^13^. Early implementations addressed this with active“dither”locking. This method introduced a small modulation on either the pump or cavity to generate an error signal for feedback control^14–16^. While effective, the residual modulation imposes a fundamental noise floor that can hinder demanding applications.

More recently, truly dither-free schemes have been demonstrated, exploiting novel error-signal generation methods that require no modulation^17,18^. Among them, the method demonstrated by Y.S. Cheng et al. in 2020 stands out for its exceptional simplicity and robustness^18^. In their work, a GHz-repetition-rate Ti:sapphire-pumped PPKTP OPO was stabilized without modulations or additional intracavity optical components, relying only on an easily implemented error signal filter with a proportional-integral (PI) controller. As an error signal, the parasitic sum frequency generation (SFG) of the pump and signal was used in that case. While dither-free stabilization schemes have proven highly effective in GHz-repetition-rate systems, scaling these concepts to MHz-rate DROPOs pumped with tens of watts, however, presents new challenges: The longer cavity lengths increase the sensitivity against acoustic, thermal, and mechanical perturbations, whereas the high power introduces unwanted thermal lensing and nonlinear phase shifts.

Here, we report on a synchronously pumped, dither-free stabilized DROPO driven by a home-built, Kerr-lens mode-locked Yb:YAG thin-disk laser. The pump laser delivers 270 fs pulses at 1030 nm with a repetition rate of 32.5 MHz and an average power of 16 W. We achieve a self-phase-locked output at the degeneracy wavelength of 2060 nm by integrating a Beta Barium Borate (BBO) crystal in a carefully dispersion-managed DROPO cavity and employing a passive error-signal spectral filter for stabilization. The resulting source provides multi-watt-level, low-noise pulses, opening new pathways for strong-field physics in the 2-$\mu$m region.

## Experimental setup

**Figure 1:**
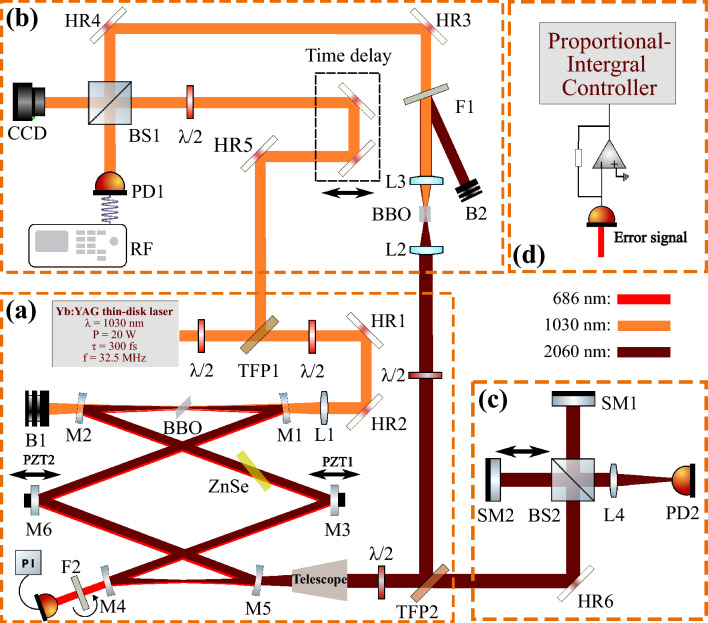
Schematics. (**a**) Experimental setup of the stabilized degenerate DROPO, synchronously pumped by a thin-disk mode-locked laser. (**b**) $f-2f$ interferometry setup to verify phase-locking between the degenerate DROPO output and the pump. (**c**) Two-photon absorption autocorrelator setup to measure the pulse durations. M1-5: cavity mirrors; HR1-6: $45^\circ$ high-reflectance mirrors; BS1-2: beam splitters; L1-3: Lenses; B1-2: Beam dumps; $\lambda$/2: half-wave plate; TFP1-2: thin-film polarizers; SM1-2: silver mirrors; RF: radio-frequency spectrum analyzer; PD1-2: photodiodes; F1-2: filters. (**d**) Schematic of the stabilization feedback loop. The parasitic SFG error signal is detected by a photodiode, pre-amplified and processed by a proportional-integral (PI) controller to generate the feedback signal for the cavity piezos.

Fig. 1(a) schematically shows the experimental setup of the DROPO, which is characterized by its bow-tie ring cavity, with approximately 9 m in length. The structure comprises two curved mirror pairs (M1-2 and M4-5) with radii of curvature of 750 mm and 300 mm. A 2 mm Brewster cut ($58.6^\circ$ at 2060 nm) BBO crystal is placed in a type-I phase-matching configuration in the larger focal point with a beam waist radius of 110 $\mu$m. The phase-matching angle of the crystal is $21.3^\circ$^19^, which is the optimal angle for 2060 nm. All the cavity mirrors are high reflectively coated from 1900 nm to 2250 nm and transmissive at the 1030 nm pump wavelength. The mirrors are designed for an incidence angle of $7^\circ$, as required by the cavity folding design. The pump power from the thin-disk laser is controlled by a $\lambda /2$ plate and a Thin-Film Polarizer (TFP). A second $\lambda /2$ plate is used to modulate the pump beam’s polarization, thereby ensuring phase-matching with the DROPO beam within the BBO crystal. The pump beam is focused into the BBO crystal using an f=400 mm lens (L1), yielding a beam waist of approximately 100 $\mu m$ ($1/e^2$ radius) at the crystal. The measured beam quality factors are $M_x^2 \approx M_y^2 \approx 1.2$, indicating that spatial walk-off and Brewster-induced astigmatism have a negligible influence on the output beam. The DROPO is operated at the near zero group delay dispersion (GDD) point realized with a 1 mm ZnSe plate. The mirror M6 is mounted on top of a slow piezoelectric transducer (PZT: Piezosystem Jena TRITOR 100), which can adjust the cavity length to a maximum of approximately 20 Hz. The mirror M3 is mounted on a fast PZT (PA44LEW Thorlabs) which can be modulated up to around 10 kHz for fine cavity-length stabilization. A 20% transmission output coupler was selected to mitigate parametric back-conversion and saturation effects observed in prior studies^20^, ensuring efficient power extraction at high pump power.

For characterization and control, three additional modules are implemented, as shown in Fig. 1. To verify phase-locking, an f-2f interferometer (Fig. 1(b)) is built by frequency-doubling the 2060 nm output with a BBO crystal cut at $21.3^\circ$ and superimposed on the original 1030 nm pump beam using a delay line and a beam splitter. Pulse duration is monitored using a home-built second-order interferometric autocorrelator based on two-photon absorption in a Si photodiode (Fig. 1(c)). Finally, the feedback loop is closed via a PI controller (Fig. 1(d)), which processes the error signal to drive the piezo actuators.

## Results and discussion

**Figure 2:**
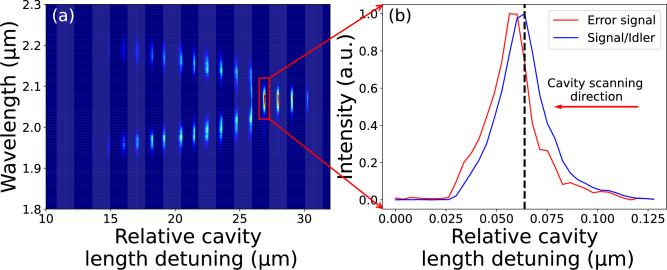
(**a**) DROPO spectra in dependence of the cavity length. (**b**) Intensity of the degenerate output at 2060 nm and the filtered parasitic SFG output serving as an error signal for the controller. Dashed line indicates the stabilized operation point.

For optimum phase locking operation, the intracavity dispersion of second and third order must be properly chosen^21^ to produce the spectral ’Y-shape’ (see Fig. 2 (a)). Namely, the GDD must be close to zero, whereas TOD should be high. Such a configuration offers enhanced benefits in terms of stabilization, exhibiting a protruding and easily identifiable self-locked region. A 1 mm ZnSe plate was placed within the cavity at Brewster’s angle to achieve near-zero GDD and a high TOD. The table  1, provides the dispersion of the materials inside the cavity. The overall resulting intracavity GDD is then approximately -33$\textrm{fs}^2$ and TOD is approximately 2604 $\textrm{fs}^3$. As a result of careful alignment of the cavity, the ’Y-shaped’ spectrum vs. detunning map is finally achieved, as shown in Fig. 2 (a). The non-degenerate and degenerate spectral areas are distinctly separated, and each resonance in degeneracy is potentially facilitating stabilization. The distance between different resonance is approximately 500 nm, and the effective length of a single degenerate resonance is approximately 20 nm.

**Table 1:** Dispersion for different materials with wavelength of 2060 nm.

Material	Effective thickness (mm)	GVD (fs$^{2}$/mm)	TOD (fs$^{3}$/mm)
BBO^19^	2	$-138$	$+1027$
ZnSe^22^	1	$+247$	$+316$
Air$^\textit{*}$^23^	9224	$6.67 \times 10^{-3}$	$2.54 \times 10^{-2}$

$^\textit{*}$ Calculated under laboratory conditions: temperature = 20$^\circ$C, pressure = 101,325 Pa, and relative humidity = 11%

As demonstrated in previous studies^13,24^, degenerate operation of the DROPO ensures phase coherence between the signal and the pump; low-power (82 mW), short-cavity (2 m) implementations operated at degeneracy remained stable for hours without feedback^25^. Our long cavity with over ten-watt pumping is inherently much more sensitive: the same length fluctuation produces a larger absolute path change and integrates more air path and mechanical fluctuations. As a result, the system quickly slips out of phase-lock. Active stabilization is therefore essential to enable a long-cavity, multi-watt degenerate DROPO to operate as a functional source.

**Figure 3:**
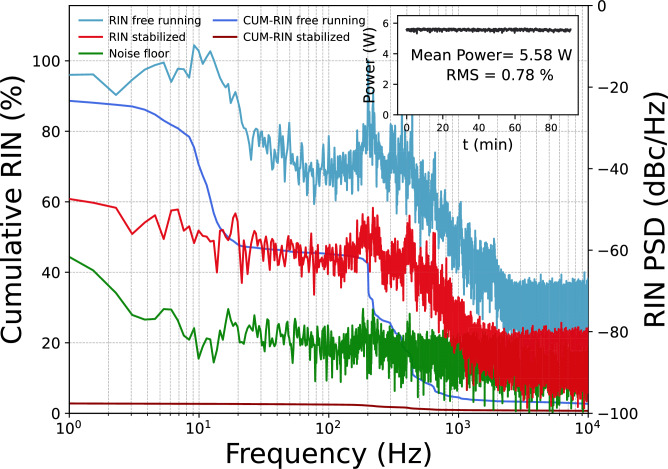
RIN and the cumulative RIN of the signal output from the DROPO for free running (blue) and stabilized operation (red). Noise floor (green) measured under the same stabilized conditions. The inset shows the long-term stability of the average output power of the DROPO over 90 minutes.

In order to achieve the active stabilization of the DROPO cavity, we implemented a PI feedback controller Fig.1(d). If a proper feedback error signal (SFG) is utilized, the P-part counteracts rapid changes that deviate from the setting point. Concurrently, the I-part is integrating the error function with respect to time, thereby compensating for the gradual drift of the cavity. The choice of a PI-only controller without a derivative module is motivated by the noise measurements in Fig. 3, which reveal that the dominant fluctuations are slow: free-running RIN is concentrated well below 100 Hz, consistent with thermally driven drift, air current perturbations, and acoustic noise. The P-part efficiently suppresses these near acoustic components, while the I-part removes residual quasi-static offsets and long term thermal drifts.

In the course of the DROPO experiment, it is possible to observe the presence of a parasitic red light from the SFG of the signal and pump. As a function of cavity length, the SFG light behaves nearly identical as the output signal, that is, both curves have maxima nearby. However, to have a sensitive error response, we need the largest possible slope in the dependence of the error signal vs cavity length around the desired operation point (see dashed line in Fig. 2 (b)). To achieve this, we used a narrow-band spectral filter (Thorlabs FBH694-10). This allowed to modify the SFG signal, rendering it a linear gradient where the DROPO signal power is maximized^18^. A slight rotation of the filter offers an additional degree of freedom to optimize the gradient. The SFG signal behind the $8^\circ$ tilted filter is shown in Fig. 2 (b). To generate the final error signal, this filtered SFG signal was detected by a photodiode and the resulting voltage was compared against a stable, adjustable DC reference within the PI controller. This enables a“side-of-fringe”lock, where the set point is adjusted to coincide with the point of maximum DROPO output power on the linear gradient of the filtered SFG signal.

The final stabilization results are presented in Fig. 3. The noise performance of the DROPO in the form of relative intensity noise (RIN) was characterized by a fast InGaAs photodiode. With recording the photodiode voltage on the oscilloscope with a sampling rate of 50 kHz for an acquisition time of 2.8 seconds per trace. The single-sided voltage power spectral density (PSD) was computed using Welch’s method. The RIN spectrum was obtained by normalizing the voltage PSD to the square of the mean DC voltage and converting to logarithmic units, yielding RIN in dBc/Hz. A noise-floor trace was recorded under identical settings and is shown in Fig. 3, it is plotted as an equivalent RIN floor referenced to the DC level of the stabilized measurement. When the cavity was operating in free-running mode, pronounced fluctuations were observed, particularly within the frequency range below 100 Hz. However, when the PI controller was engaged, the RIN noise was significantly reduced to below -50 dBc/Hz. The cumulative RIN, calculated by integrating from high frequency down to 1 Hz, the cumulative RIN is reduced from 88.9% (free running) to 2.8% (stabilized), corresponding to an improvement by a factor of 32 over the measured bandwidth. The long-term stability measurement of the output power was measured by a Thorlabs S425C-L power meter for over 90 minutes. Because the power meter has a response time of 0.6 s, the root-mean-square (RMS) measurement here represents fluctuations below 1 Hz. This result is depicted in the inset in Fig. 3. A stable output power of 5.58 W was achieved at degeneracy with a pump power of 15.8 W, which results in a conversion efficiency of 35.3%. The RMS deviation for the measurement was 0.78%. This is the maximal power of phase-locked femtosecond-scale OPO operating within the 2-$\mu$m range reported up to now, to the best of our knowledge.

**Figure 4:**
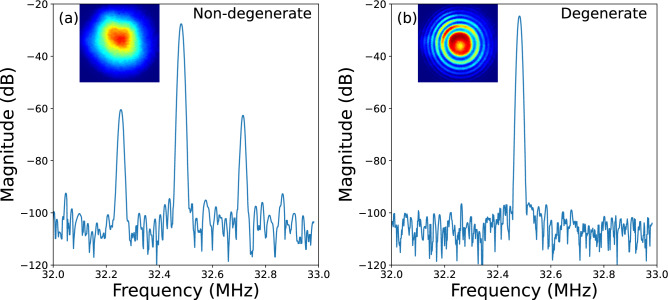
Radio-frequency spectra of DROPO output in (**a**) non-degenerate and (**b**) degenerate state. Inset shows the spatial fringe patterns observed in each state.

**Figure 5:**
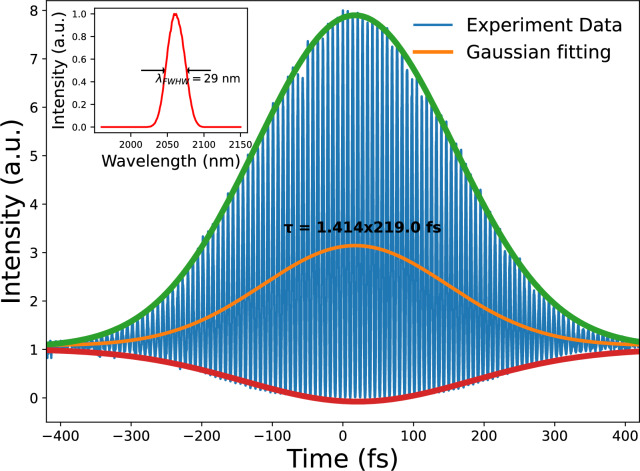
Second-order autocorrelation trace measured by two-photon detector, as a function of delay time. Insert: Corresponding spectrum of the degenerate output of DROPO.

The phase coherence between the pump and the signal was verified using the f-2f interferometer setup described previously Fig. 1(b). The spatial interference pattern between the two beams were recorded by a CCD camera and are displayed as an inset of Fig. 4. Once DROPO was stabilized in degenerate operation, a stable fringe pattern was observed on the camera. Depending on the relative alignment and mode matching of the two arms, the interference pattern can appear either as linear fringes or as concentric rings. In our setup we intentionally keep alignment freedom between the two beam paths to optimize fringe visibility and contrast for visualization. If the cavity were detuned from degenerate to non-degenerate operation, the spatial interference pattern disappeared. Furthermore, a radio frequency (RF) measurements were performed to further confirm the phase relationship of the two beams. In Fig. 4, the RF traces for both the non-degenerate (non-stabilized) and degenerate (stabilized) cases are presented. Satellite peaks at 32.2 MHz and 32.7 MHz can be observed, respectively, with the fundamental peak of 32.5 MHz. Upon stabilization of the DROPO cavity to degeneracy, the satellite peaks vanished and a clean solitary peak at the fundamental repetition rate persisted, as shown in Fig. 4(b). Furthermore, the long-term measurements revealed a persistent presence of the spatial interference pattern, indicating long-term phase-locked stability. After stabilization of our DROPO, we can reliably measure the pulse duration. The duration of the DROPO 2060 nm laser pulse is measured by a second-order interferometric autocorrelator, based on a Si photodiode serving as a two-photon detector, as shown in Fig. 1(c). The results of the autocorrelation setup are displayed in Fig.  5. The full width at half maximum (FWHM) of intensity autocorrelation was measured to be 310 fs. Accordingly, the pulse duration of 2060 nm pulse can be calculated to be 219 fs, assuming a Gaussian pulse. The Fig. 5 inset shows the spectrum of the stabilized degenerate of DROPO with a FWHM of 29 nm, indicating a Fourier-transform-limited pulse duration of 215 fs. As previously described, the GDD was managed throughout the entire cavity close to zero. This finding is consistent with transform-limited and unchirped pulsing.

## Conclusion

In summary, we demonstrated the possibility of using the dither-free stabilization method to stabilize a mode-locked thin-disk pumped DROPO at degeneracy. A high output power of 5.58 W was achieved at 2060 nm, with a stable phase-locked relationship with a 1030 nm pump laser. Within the available pump power range, no saturation or thermally induced power limitation were observed, indicating that higher output power should be possible with a more powerful pump source and optimized output coupling. Such scaling would facilitate strong-field experiments that necessitate phase-locked multi-color interaction for field shaping, such as Brunel radiation^10^.

## Data Availability

The data that support the findings of this study are available from the corresponding author upon reasonable request.

## Declarations

### Additional information

**Correspondence** and requests for materials should be addressed to H.R. **Reprints and permissions information** is available at http://www.nature.com/reprints.
